# Age-specific patterns of breast cancer in Nigerian women unraveled through histological analysis

**DOI:** 10.1038/s41598-025-28685-0

**Published:** 2025-11-29

**Authors:** Magdalene Eno Effiong, Shalom Nwodo Chinedu, Israel Sunmola Afolabi, Kevin Nwabueze Ezike, Emmanuel Eyitayo Oguntebi, Oluwasesan Adelowo Abdul, Izuchukwu Benerdin Achusi, Tolulope Aanuoluwapo Benye, Mercy Bella-Omunagbe, Peace Nzubechukwu Ogbodo

**Affiliations:** 1 Department of Biochemistry, College of Science and Technology, Covenant University, Canaanland, PMB 1023, Ota, Ogun Nigeria; 2 Covenant Applied Informatics and Communication Africa Centre of Excellence (CApIC-ACE), Ota, Nigeria; 3 Covenant University Public Health and Wellbeing Research Cluster (CUPHWERC), Covenant University, Canaanland, PMB 1023, Ota, Ogun Nigeria; 4 Department of Histopathology, Asokoro District Hospital, Abuja, FCT Nigeria; 5 Department of Anatomic Pathology and Forensic Medicine, Nile University of Nigeria, Abuja, FCT Nigeria; 6 Department of Pathology and Forensic Medicine, University of Abuja, Gwagwalada, FCT Nigeria; 7 Department of Histopathology, University of Abuja Teaching Hospital, Gwagwalada, FCT Nigeria; 8 Department of Anatomic Pathology, Federal Medical Centre, Jabi, Abuja, FCT Nigeria

**Keywords:** Age patterns, Breast cancer, Lesions, Malignant, Benign, Nigeria, Sub-Saharan africa, Epidemiology, Biochemistry, Cancer, Molecular biology, Diseases, Health care, Medical research, Oncology, Pathogenesis, Risk factors

## Abstract

Sub-Saharan African women face a high burden of breast cancer, influenced by genetic and lifestyle factors. However, the lack of comprehensive, age-stratified data hinders the identification of risk factors and the development of effective, population-specific interventions. This study aimed to assess age-related variations in breast cancer prevalence among Nigerian women, providing insight into associated risk factors and disease trends. A retrospective review of 3,263 breast histopathology records (9.46% of total from 2015 to 2023) was conducted. Lesions—benign and malignant—were analyzed across five age groups: children and adolescents (0–19), young adults (20–39), middle-aged (40–59), higher-aged (60–79), and elderly (≥ 80), using MS Excel and GraphPad Prism 8.0. Statistical comparisons were performed by age and lesion type. Most cases were in young adults (45.97%) and middle-aged women (33.83%). The left breast was more commonly affected (46.86%) and had higher malignancy rates than the right (44.41%) or bilateral lesions (7.20%). Benign lesions were predominant (56.76%), especially among young adults (57.34%). Malignancy incidence increased with age, peaking in middle-aged women (53.30%). Fibroadenoma was the most frequent benign lesion in children and adolescents and young adults, while fibrosis predominated in middle age. Invasive ductal carcinoma (IDC) was the leading malignant subtype, with a sharp rise by 2023—particularly among middle-aged (172 cases) and young adult women (71 cases). Among 339 immunohistochemically profiled cases, triple-negative breast cancer (TNBC; 42.77%) and ER+/PR+ tumors (36.87%) were most common. TNBC was the only subtype detected in children and adolescents. Middle-aged women bore the highest burden of all subtypes, with a marked increase in TNBC and ER+/PR+ cases in 2023. The rising incidence of aggressive subtypes, particularly TNBC, highlights the need for enhanced molecular diagnostics and personalized therapies. Age-specific trends reinforce the urgency for targeted screening, especially for young and middle-aged Nigerian women.

**Supplementary Information:**

The online version contains supplementary material available at 10.1038/s41598-025-28685-0.

## Introduction

Breast cancer remains a significant public health challenge globally, particularly in developing countries like Nigeria, with high incidence and mortality rate^1,2^. The incidence of breast cancer in Nigeria has been reported to be on the rise, with significant regional variations^3,4^. Myriad of factors, including lesion types, demographic influences, and risk factors, are said to influence the incidence and progression of breast cancer^5,6^. Among the risk factors of breast cancer identified in Nigerian populations are genetic predispositions, reproductive history, and lifestyle factors^7,8^. Family history of breast cancer is also a significant risk factor as studies have shown that women with a first-degree relative diagnosed with breast cancer have a higher likelihood of developing the disease^9,10^. Other factors that have been associated with increased breast cancer risk include age, early menarche, late menopause, nulliparity, and the use of hormonal contraceptives and many more^11–13^.

Breast cancer accounts for about 50% of all cancers in Nigerian women, making it the most common malignancy among females^14^. Studies by Nwafor et al.^15^, Raphael et al.^16^, Edegbe et al.^17^, Rioki et al.^18^, Okafor et al.^19^, indicate that fibroadenomas are the most common type of benign lesion diagnosed in Nigeria. Among the malignant lesions, Invasive Ductal carcinoma (IDC) is the most frequently diagnosed, accounting for over 80% of cases^20^. Other notable subtypes include ductal carcinoma in situ (DCIS) and lobular carcinoma, although these are less frequently reported^21^. Moreover, molecular subtyping has gained attention in recent years, particularly with the classification of lesions into hormone receptor-positive (ER+/PR+), HER2-positive, and triple-negative breast cancer (TNBC)^22^. Studies have shown that TNBC is prevalent among younger women and is associated with poorer prognoses^23^. The demographic distribution of these molecular subtypes highlights the need for targeted therapies and personalized treatment approaches.

Breast cancer is a multifaceted disease influenced by various lifestyle and nutritional habits, which account for over 50% of cases through acquired genetic mutations^24,25^. Factors such as the consumption of red and smoked meats, alcohol, carbonated beverages, smoking, and antibiotic use are prominent contributors^7,25^. Previous research by Effiong et al.^10^ highlighted that middle-aged Nigerian women, aged 40–60 years, face the highest breast cancer risks due to their lifestyle and dietary patterns. However, a comprehensive study of how breast cancer prevalence varies across all age groups is essential to identify population-specific risk factors and target interventions effectively. This knowledge is particularly important in Nigeria, where cultural, socioeconomic, and dietary differences across age groups significantly impact health outcomes.

Building on Effiong et al.‘s^7,11^ findings, analyzing the variability in breast cancer prevalence among age groups provides vital insights into the relationship between risk variables and disease prevalence. This approach will not only illuminate the age-specific burden of breast cancer but also facilitate the development of tailored prevention, early detection, and treatment strategies. By correlating risk levels with prevalence, this research aims to address gaps in current cancer control efforts, enabling the design of age-targeted public health policies and interventions. Such measures are crucial for reducing the overall breast cancer burden and improving survival rates in Nigerian women, aligning with global efforts to combat cancer through precision public health approaches^26,27^.

## Methods

### Sampling technique

**Fig. 1:**
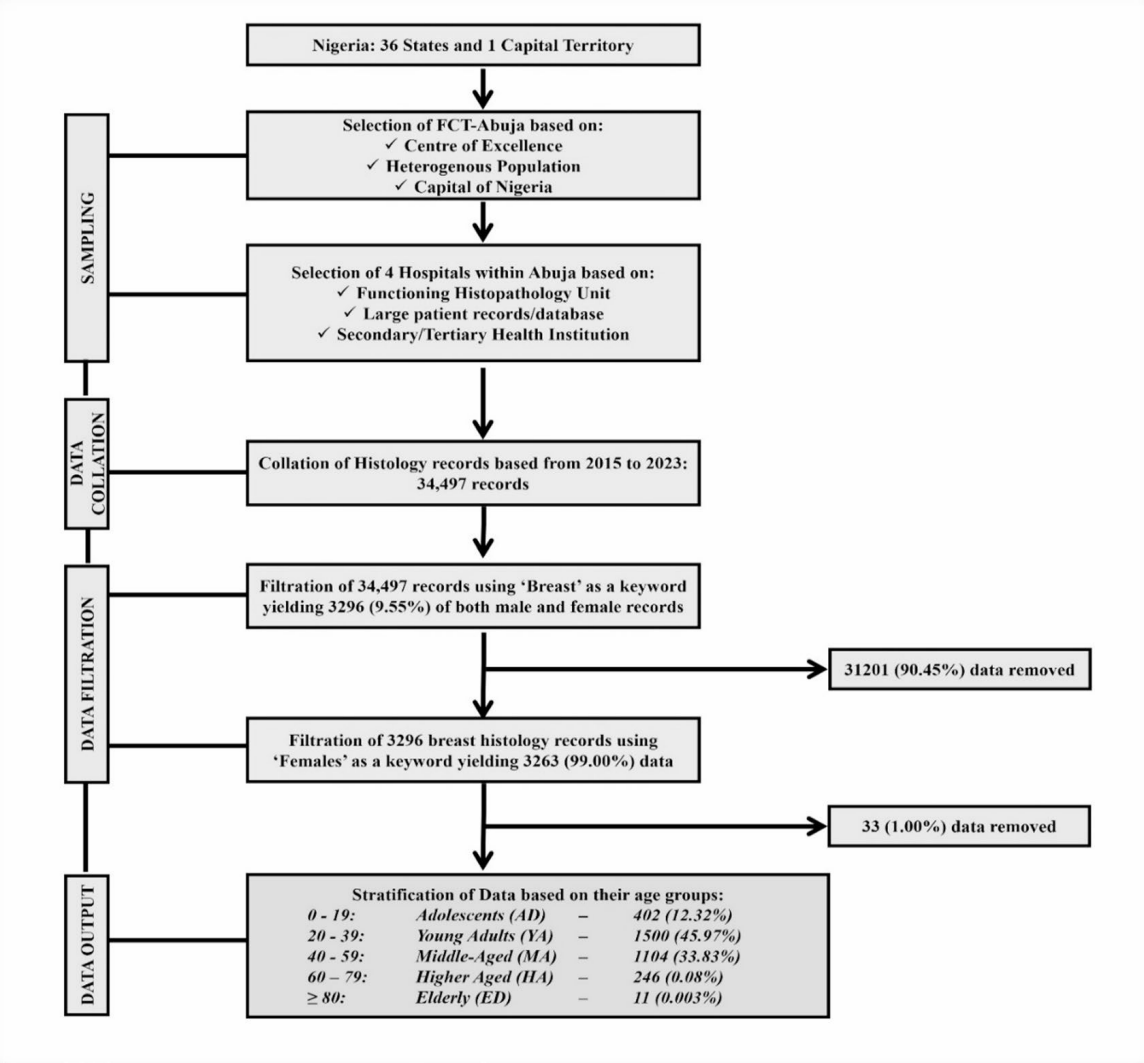
Data collection flow chart.

A retrospective study was conducted in four secondary-tertiary hospitals in Abuja, Federal Capital Territory, Nigeria from 2015 to 2023 (Fig. 1). Case notes of histologically examined breast lesions categorized as benign and malignant were identified using the health information records from the Anatomic pathology unit of the hospitals. The benign lesion were categorized as controls while the malignant lesions were categorized as case. The molecular classification into the subtypes of breast cancer was obtained based on the recorded immunohistochemical evaluation of the breast lesions carried out by the histopathological unit in the hospital. The variables of the study include biodata, demographics, lump duration, menopausal status, parity, type of diagnosis, among others.

### Sample size determination

The sample size for this study was determined through a multi-stage, systematic selection and filtration process (Fig. 1). Initially, the Federal Capital Territory (FCT–Abuja) was purposively selected due to its status as a “Centre of Excellence,” its heterogeneous population, and its role as the capital of Nigeria. Within FCT–Abuja, four hospitals were purposively chosen based on the following criteria: (i) presence of a functioning histopathology unit, (ii) availability of a large patient record/database, and (iii) designation as secondary or tertiary health institutions.

All histology records from these hospitals spanning 2015 to 2023 were collated, yielding an initial dataset of 34,497 records. A stepwise filtration was then applied: first, 3,296 records (9.55%) containing “Breast” as a keyword were extracted; subsequently, 3,263 records (99.00%) specifically mentioning “Females” were identified. This final dataset of 3,263 female breast histology records constituted the study’s sample size, which was stratified by age for analysis.

### Inclusion criteria

Case reports of histologically examined breast lesions (malignant and benign) of female Nigerian patients recorded within the period of 2015 to 2023 in the selected health institutions in Abuja, FCT, Nigeria. The data was accessed from 2023 to 2024.

### Exclusion criteria

Records without histological diagnosis and sufficient data were excluded.

### Ethical consideration

The research team obtained approval from the management of Covenant University to carry out the study. The Covenant Health Research Ethics Committee (CHREC) and National Health Research Ethics Committee of Nigeria (NHREC) granted ethical approval for this study under the reference number CU/HREC/EME/204/23 and NHREC/01/01/2007-16/08/2023 respectively. Ethical approval the respective hospitals were also obtained with the reference numbers: NHA/EC/045/2023; FMCABJ/HREC/2023/093; FCT/UATH/HREC/11,015 and FCTA/HHSS/HMB/ADH/144/23 which covered a one year duration from 2023 to 2024. Due to the retrospective nature of the study, the need to obtain the informed consent was waived by the University of Abuja Teaching Hospital ethical committee, Federal Medical Centre Abuja ethical committee, Federal Capital Territory Asokoro ethical committee. This study strictly adhered to the ethical norm associated with human research. The study team did not have access to any information could identify individual participants during or after data collection.

### Statistical analysis

Data were collated and analyzed using Microsoft Excel (Microsoft Corporation, USA) and GraphPad Prism version 8.0 (GraphPad Software, USA). Descriptive statistics were applied, with categorical variables summarized as frequencies and percentages. Continuous variables, where applicable, were expressed as means ± standard deviation (SD).

Comparisons across age strata and clinical subgroups were performed using Chi-square tests or Fisher’s exact test for categorical data. Parametric (Student’s t-test, one-way ANOVA with post hoc Tukey’s test) or non-parametric equivalents (Mann–Whitney U test, Kruskal–Wallis test) were employed depending on the distribution of continuous variables, which was assessed using the Shapiro–Wilk test.

Regression and correlation analyses were conducted to evaluate associations between variables. Odds ratios (OR) with 95% confidence intervals (CI) were calculated to assess the strength of associations, while multivariate logistic regression was applied to adjust for potential confounders such as age and hospital source. Statistical significance was set at a two-tailed *p* < 0.05.

## Results

### Sociodemographic characteristics

A total of 3263 records were examined which consisted of 9.46% of the total breast histology records from the period of 2015 to 2023, comprising 1411 malignant and 1,852 benign lesions. All the records (100%) examined were females (Supplementary Table 1). The data of breast tissues obtained were analyzed and divided into five age groups, consisting of: Children and adolescents (0–19 years)—402 (12.32%), young adults (20–39 years)—1500 (45.97%), middle aged (40–59 years)—1104 (33.83%), higher aged (60–79 years)—246 (7.54%) and elderly (80 and above)—11 (0.34%). Several clinical and demographic variables were examined to determine their association with breast malignancy (Table 1).

Laterality of breast lesions showed a significant association with malignancy. Lesions on the left breast were significantly more likely to be malignant compared to bilateral lesions (OR: 2.77; 95% CI: 1.90–4.07; *p* < 0.001). Similarly, right-sided lesions were also associated with higher odds of malignancy (OR: 2.47; 95% CI: 1.70–3.60; *p* < 0.001). In contrast, bilateral lesions were more frequently benign. Analysis of geographical distribution indicated no statistically significant difference in malignancy risk across regions. Although patients from the east and west had slightly higher frequencies of malignancy, these associations were not statistically significant (e.g., south vs. north: OR: 1.00; 95% CI: 0.81–1.23; *p* = 0.99).

Family history of breast cancer, when reported, did not significantly influence malignancy risk. The odds of malignancy in patients with a positive family history were not statistically different from those without (OR: 0.85; 95% CI: 0.32–2.29; *p* = 0.74), and the majority of patients did not state their family history. Duration of breast lump presence showed some significant associations. Lesions that had been present for 1–5 years had higher odds of being malignant compared to those of less than one-year duration (OR: 1.27; 95% CI: 1.01–1.61; *p* = 0.041). However, longer durations (≥ 6 years) did not show consistent or significant associations, possibly due to smaller sample sizes. Parity was significantly associated with malignancy. Nulliparous women (0 parity years) had significantly lower odds of malignancy and were more likely to have benign lesions (OR: 0.07; 95% CI: 0.05–0.10; *p* < 0.001). Conversely, women with ≥ 5 years of parity had over four times the odds of developing malignancy compared to nulliparous women (OR: 4.11; 95% CI: 2.58–6.55; *p* < 0.001).

Among the 1,411 malignant lesions analyzed, 709 were graded (Grades 1–3) and 702 were unspecified; Grade 2 was most frequent (35.3%), followed by Grade 3 (12.8%), while Grade 1 was rare (2.1%) (Supplementary Table 3). The middle-aged group accounted for over half of cases (53.3%), with Grade 2 predominating in both middle-aged (61.9%) and young adults (63.2%), whereas higher-aged women showed a more balanced grade distribution. Chi-square analysis confirmed a significant association between age and grade (χ² = 44.06, df = 8, *p* < 0.001), and logistic regression indicated that middle-aged women had the highest odds of Grade 2 lesions (OR = 1.27, 95% CI: 0.13–12.35), compared with lower odds in young adults and higher-aged women, though estimates were imprecise in children and adolescents and elderly due to small sample sizes. Collectively, these findings demonstrate marked age-specific variation in nuclear grading of malignant lesions. Mastectomy was exclusively performed in malignant cases and was significantly associated with malignancy (OR: 443.0; 95% CI: 27.4–7168.2; *p* < 0.001). In contrast, excisional biopsy was more frequently used for benign lesions (OR: 0.36; 95% CI: 0.30–0.43; *p* < 0.001).

With respect to menopausal status, malignancy was significantly more prevalent among menopausal and postmenopausal women. Postmenopausal women had almost five times the odds of having malignant lesions compared to premenopausal women (OR: 4.82; 95% CI: 3.33–6.97; *p* < 0.001), and similar associations were seen with menopausal women (OR: 2.73; 95% CI: 2.21–3.38; *p* < 0.001). Only 10.70% (151 cases) of the malignant lesions had metastasis or history of chemotherapy.

**Table 1:** Biodata and sociodemographic characteristics.

		Malignant	Benign	Odds ratio	95% CI lower	95% CI upper	*P*-value	Significance
A	Breast location							
	Left	710	819	0.99	0.89	1.1	0.9146	Not significant
	Right	650	799	0.93	0.83	1.03	0.1696	Not significant
	Bilateral	32	203	0.18	0.12	0.26	< 0.0001	Significant ↓
	Not stated	19	31	–	–	–	–	–
B	Region							
	North	443	660	0.76	0.67	0.86	< 0.0001	Significant ↓
	South	221	313	0.8	0.68	0.96	0.0150	Significant ↓
	East	488	573	0.97	0.86	1.1	0.7030	Not significant
	West	259	306	0.97	0.82	1.15	0.7320	Not significant
C	Family history							
	Yes	12	10	1.38	0.59	3.19	0.5240	Not significant
	No	11	7	1.8	0.7	4.65	0.2440	Not significant
	Not stated	1388	1835	–	–	–	–	–
D	Lump duration							
	Less than 1 year	204	265	0.88	0.73	1.06	0.1890	Not significant
	1–5 years	180	276	0.74	0.61	0.9	0.0020	Significant ↓
	6–10 years	14	37	0.43	0.23	0.8	0.0070	Significant ↓
	11–15 years	3	7	–	–	–	–	–
	16–20 years	4	5	–	–	–	–	–
	21–25 years	1	1	–	–	–	–	–
	26–30 years	1	0	–	–	–	–	–
	Not stated	1004	1261	–	–	–	–	–
E	Parity							
	0 year	56	661	0.09	0.07	0.12	< 0.0001	Highly significant ↓
	1–2 years	42	50	0.96	0.64	1.45	0.9170	Not significant
	3–4 years	65	27	2.77	1.77	4.35	< 0.0001	Significant ↑
	≥ 5 years	56	16	4.03	2.31	7.03	< 0.0001	Significant ↑
	Not stated	1192	1098					
G	Method of sample collection							
	Incision	428	189	2.68	2.26	3.19	< 0.0001	Significant ↑
	Excision	645	1474	0.46	0.42	0.51	< 0.0001	Significant ↓
	Mastectomy	151	0	–	–	–	–	–
	Tissue blocks	7	0	–	–	–	–	–
	Not stated	180	171	–	–	–	–	–
H	Menopausal state							
	Premenopausal	443	450	1.14	0.99	1.3	0.0680	Borderline
	Menopausal	752	359	2.54	2.23	2.89	< 0.0001	Significant ↑
	Postmenopausal	216	43	5.89	4.24	8.18	< 0.0001	Highly significant ↑

### Trends in the occurrence of malignant and benign lesions from 2015 to 2023

**Fig. 2:**
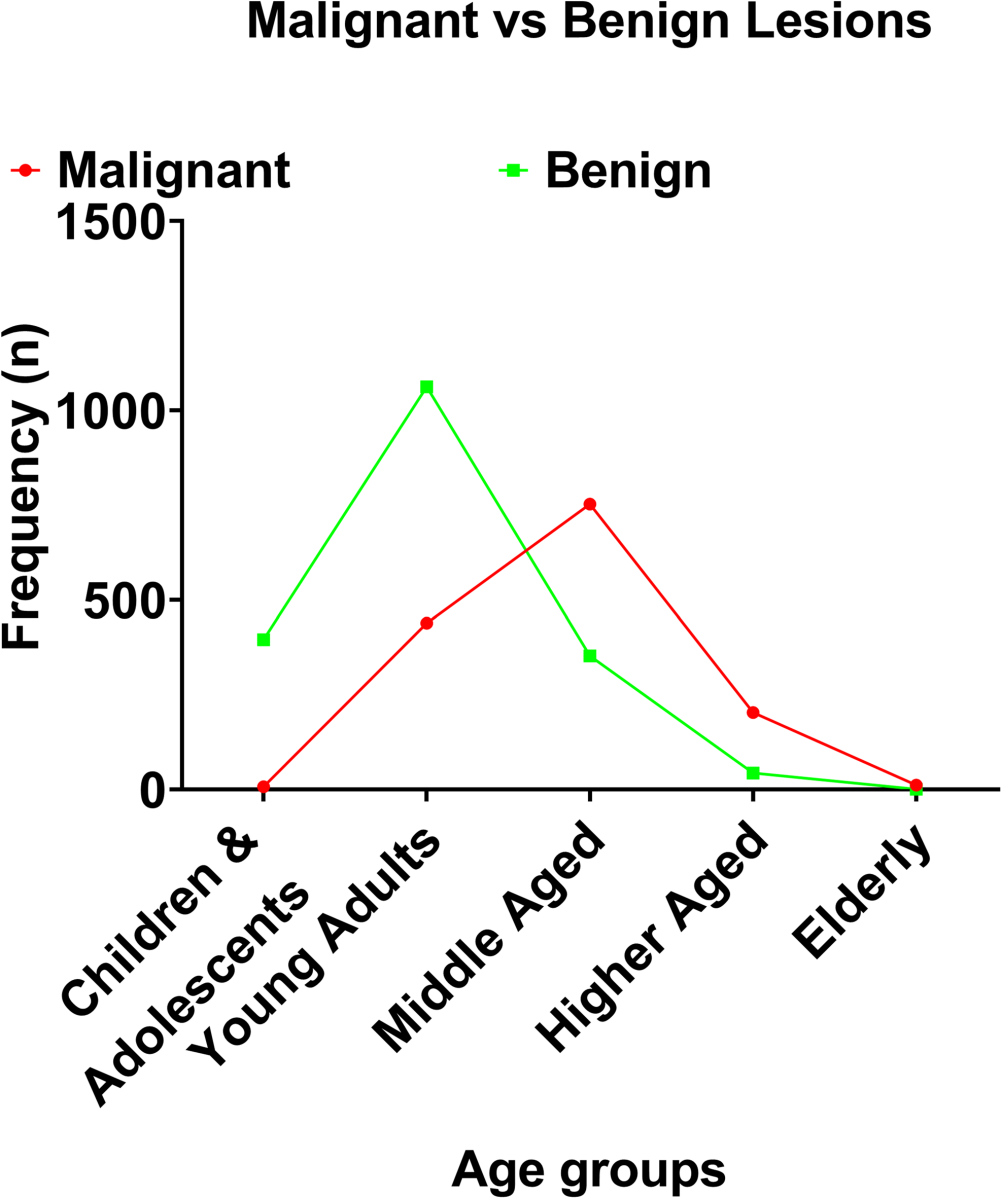
Occurrence of malignant and benign lesions across age groups of Nigerian women.

A total of 3,263 female breast histology records were analyzed across five age strata: children and adolescents, young adults, middle-aged, higher-aged, and elderly. The distribution of malignant and benign lesions varied significantly across age groups (Fig. 2; Supplementary Tables 1 and 2). Benign lesions were more prevalent in younger women, particularly among young adults, who accounted for 1,062 cases (57.3%). In contrast, malignant lesions increased progressively with age, becoming the dominant lesion type among middle-aged women, with 752 cases (53.3%), and continuing to predominate in the higher-aged and elderly groups.

**Fig. 3:**
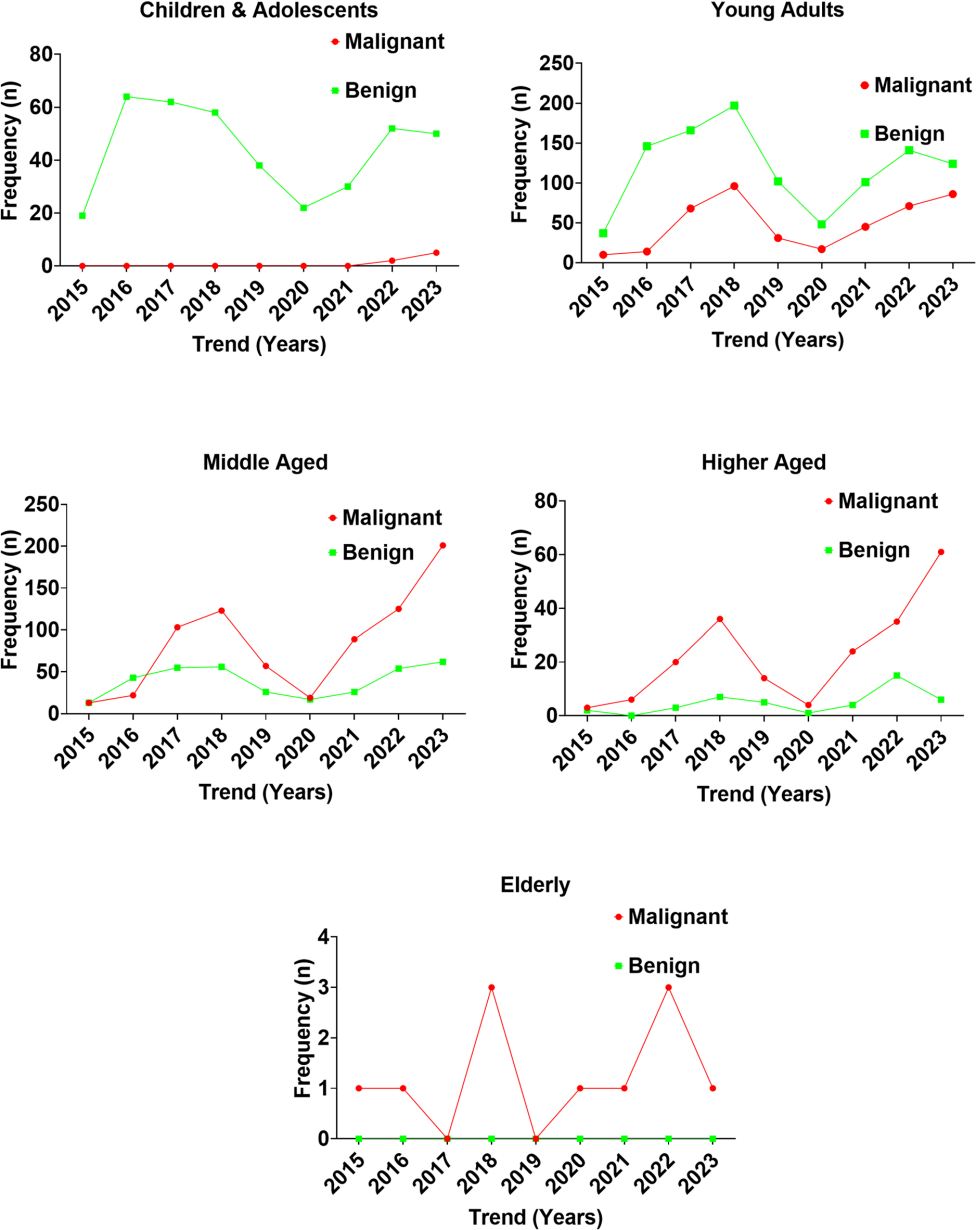
Trends in benign and malignant lesions across age groups of Nigerian women from 2015 to 2023.

Figure 3 further illustrates the temporal distribution of benign and malignant lesions across age groups between 2015 and 2023 (Supplementary Table 2). Among children and adolescents (≤ 19 years), both lesion types remained rare throughout the study period. Malignant cases never exceeded 10 annually, while benign cases fluctuated modestly but remained below 70 per year, suggesting a low burden of breast pathology in this age category. In young adults (20–39 years), lesion frequency increased, with benign lesions peaking around 2017–2018 at nearly 200 cases before declining to ~ 100 cases in 2023. Malignant lesions showed a steady upward trend, rising from fewer than 25 cases in 2015 to ~ 125 cases by 2023.

The middle-aged group (40–59 years) demonstrated a marked transition. Benign lesions peaked at ~ 125 cases during 2017–2018 but declined to ~ 75 cases by 2023. In contrast, malignant lesions rose sharply from fewer than 25 cases in 2015 to ~ 225 cases in 2023, signifying a critical age range where malignancies surpassed benign lesions in frequency. Among higher-aged women (60–79 years), malignant lesions continued to increase, rising from a low baseline in 2015 to nearly 75 cases in 2023, whereas benign lesions remained consistently lower, averaging ~ 25 cases annually by the end of the study period. The elderly group (≥ 80 years) recorded the lowest overall incidence of breast pathology, with annual cases of both malignant and benign lesions rarely exceeding three.

The Chi-square test confirmed a highly significant association between age group and histological diagnosis (χ² = 849.99, df = 4, *p* < 0.0001). Logistic regression analyses, using children and adolescents as the reference group, revealed a progressive increase in the odds of malignancy with advancing age. Young adults were approximately 23 times more likely to present with malignant lesions compared to children and adolescents (OR = 23.27, 95% CI: 10.93–49.54). The odds rose sharply in middle-aged women (OR = 120.55, 95% CI: 56.49–257.26) and higher-aged women (OR = 266.40, 95% CI: 117.73–602.78). In the elderly group, all cases were malignant, resulting in an infinite odds ratio.

### Trend in benign breast lesions prevalence across age-groups

**Fig. 4:**
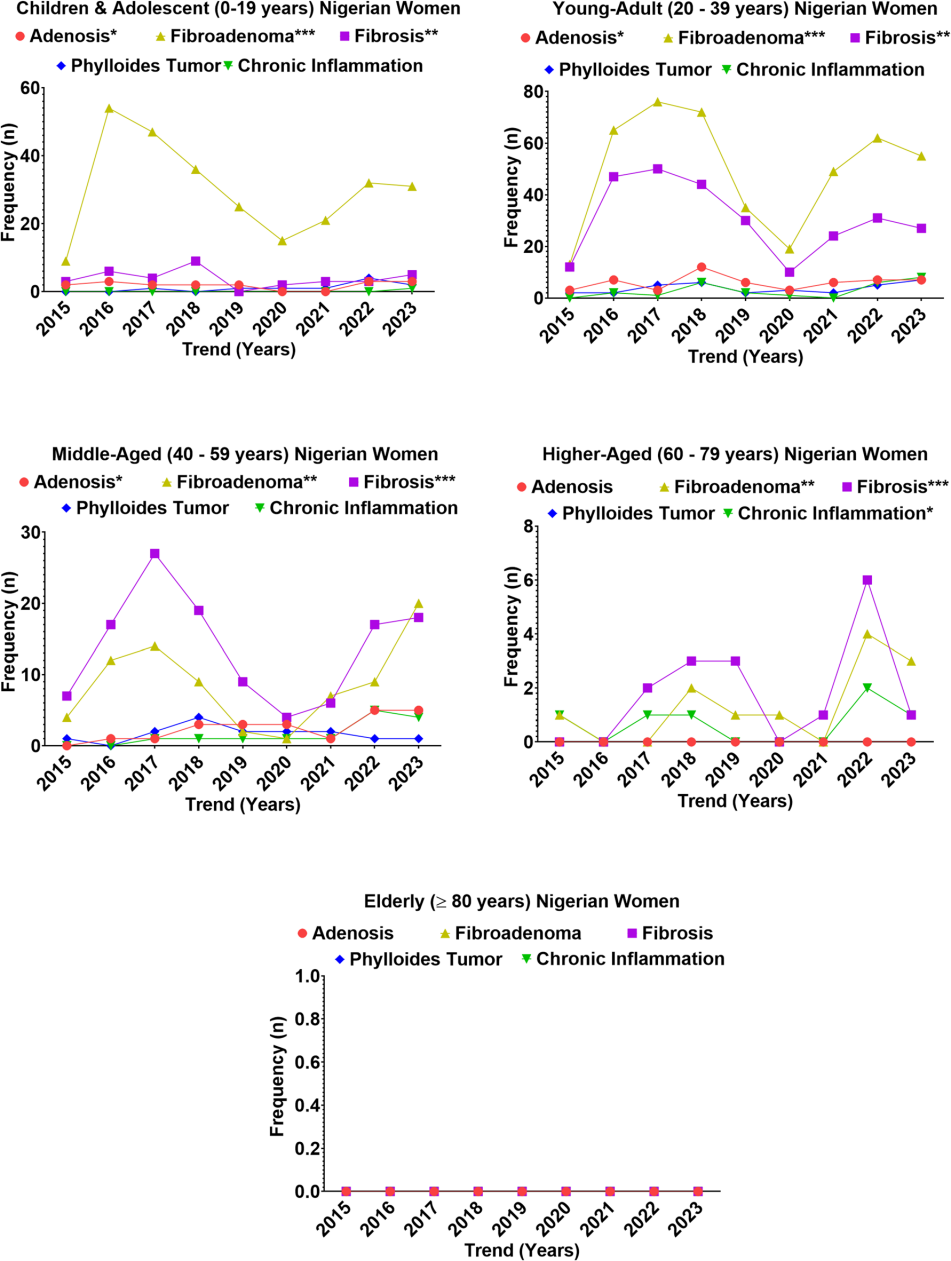
Trends in the occurrence of benign breast lesions among age groups of Nigerian women from 2015 to 2023.

Various types of benign breast lesions were evaluated from institutional records, comprising 56.8% of all breast cases during the study period (Fig. 4, Supplementary Table 4). Descriptive analysis showed that fibroadenoma was the predominant benign lesion, accounting for the majority of cases across age groups (*n* = 806, 61.6%). It was particularly concentrated among children and adolescents (270 cases, 33.5%) and young adults (446 cases, 55.4%), with middle-aged and higher-aged women contributing far fewer cases (78 and 12 respectively). Fibrosis ranked second in frequency (*n* = 450, 34.4%), again heavily concentrated in young adults (275 cases, 61.1%) and middle-aged women (124 cases, 27.6%), while adenosis was rare overall (*n* = 93, 7.1%) and occurred sporadically across younger age categories. Chronic inflammatory lesions (*n* = 47, 3.6%) and benign phylloides tumors (*n* = 69, 5.3%) were uncommon, with phylloides peaking in young adults (34 cases, 49.3%) and middle-aged women (15 cases, 21.7%), while higher-aged women contributed isolated cases. Notably, no benign lesions were recorded in elderly patients across the nine-year period.

Temporal trends revealed distinct peaks: fibroadenoma surged in children and adolescents and young adults between 2016 and 2018, while fibrosis peaked among young adults in 2016–2017 (*n* = 97). Adenosis showed small but recurrent rises after 2018, particularly in middle-aged women. Chronic inflammatory lesions were observed mainly from 2018 onwards, increasing among middle-aged and higher-aged groups. Phylloides tumors were sporadic but showed slight clustering in 2017–2018 and 2022–2023, mostly in young and middle-aged women.

Inferential analysis demonstrated a significant association between age group and lesion subtype (χ² = 190.42, df = 12, *p* < 0.001). Logistic regression, adjusting for year of diagnosis, confirmed that children and adolescents and young adults had markedly higher odds of presenting with fibroadenoma compared to women ≥ 40 years (OR = 5.8, 95% CI: 3.2–10.4, *p* < 0.001). Conversely, middle-aged women had significantly higher odds of developing fibrosis compared with children and adolescents (OR = 3.1, 95% CI: 1.7–5.6, *p* = 0.002). Chronic inflammation and phylloides lesions, although less frequent, showed a modest age-related gradient: women ≥ 40 years had a 2.4-fold increased odds of chronic inflammation compared with those < 30 years (OR = 2.4, 95% CI: 1.1–5.1, *p* = 0.029). A chi-square test for linear trend confirmed a statistically significant rise in fibroadenoma and fibrosis in younger age groups over time (*p* < 0.001), whereas adenosis, chronic inflammation, and phylloides did not exhibit consistent temporal trends. These results highlight a distinct age-related transition in the types of benign breast lesions, moving from a clear dominance of fibroadenoma in younger women toward fibrosis and other benign lesions in older age groups.

### Distribution of malignant breast lesions across age groups of Nigerian women (2015–2023)

**Fig. 5:**
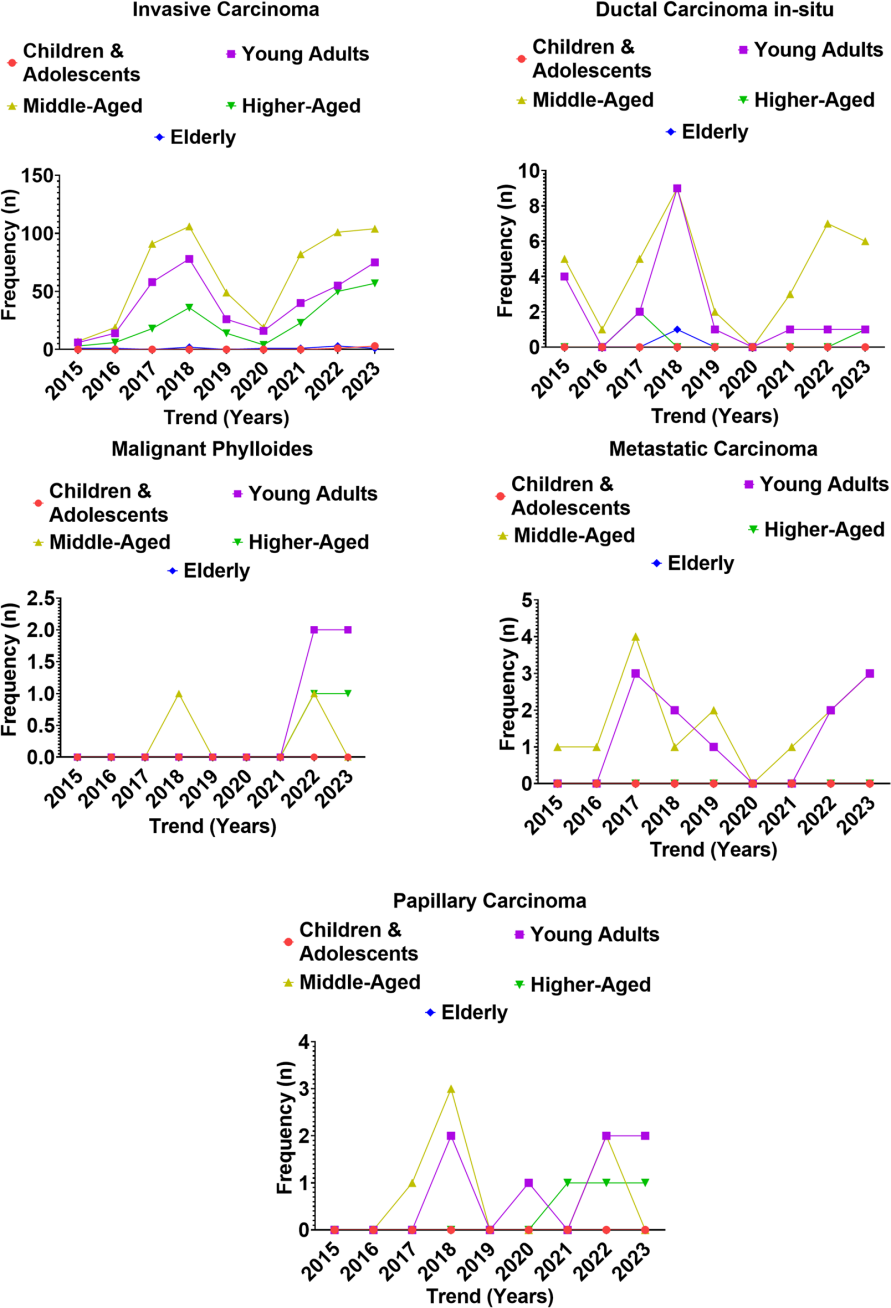
Trend in malignant lesions across age groups of Nigerian women from 2015 to 2023.

**Fig. 6:**
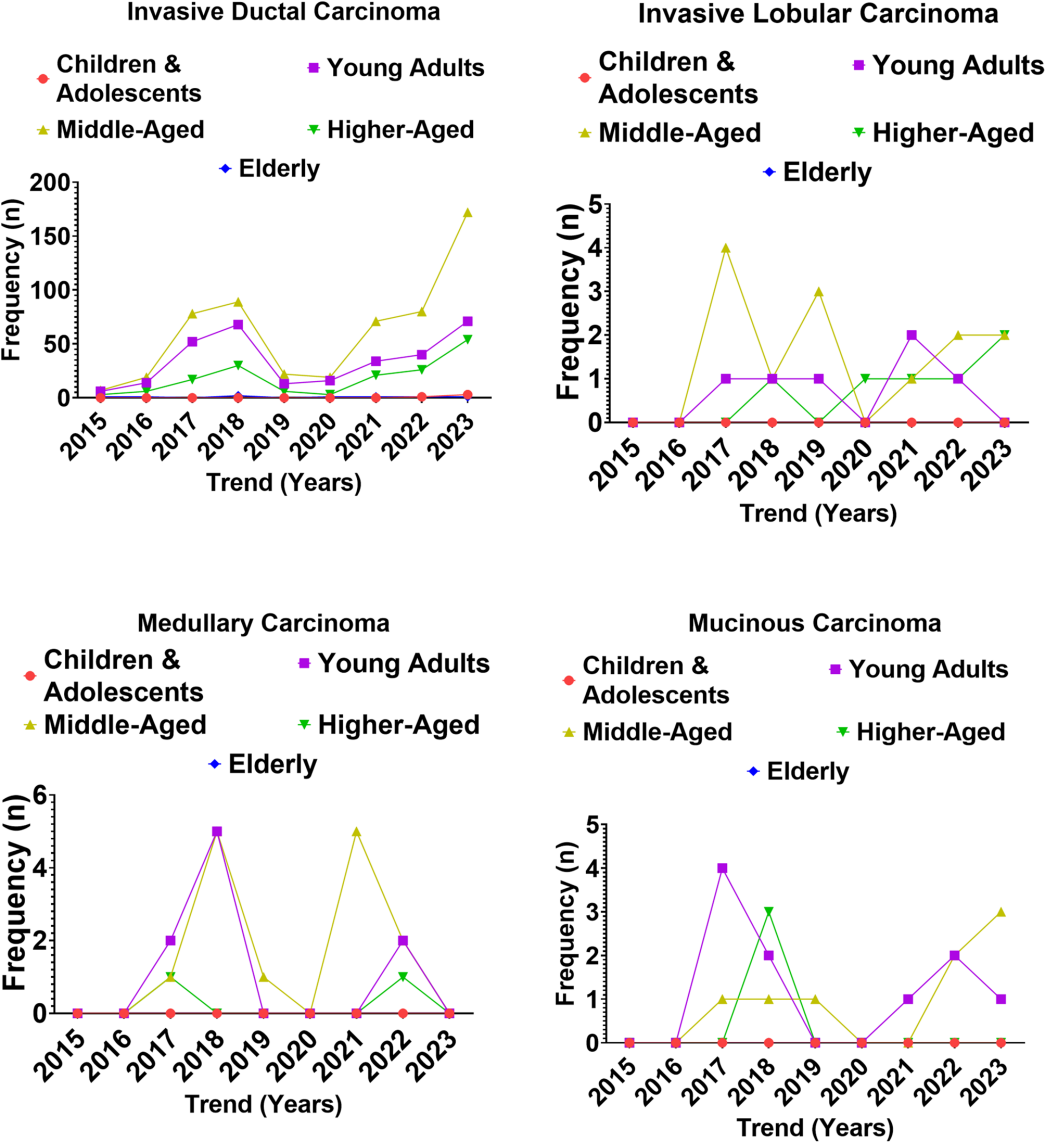
Variation in the occurrence of malignant lesions across age groups of Nigerian women from 2015 to 2023.

Analysis of malignant breast lesions from 2015 to 2023 demonstrated clear age- and subtype-specific patterns (Figs. 5 and 6, Supplementary Table 5). A total of 1,241 malignant breast lesions were recorded between 2015 and 2023, comprising invasive carcinoma (*n* = 1,171; 94.4%), ductal carcinoma in situ (DCIS; *n* = 39; 3.1%), metastatic carcinoma (*n* = 14; 1.1%), papillary carcinoma (*n* = 10; 0.8%), and malignant phylloides (*n* = 7; 0.6%). Invasive carcinoma was the predominant histological subtype across all age groups and years, whereas other malignancies were relatively rare. Age-specific variation was evident. Invasive carcinoma was most frequent among middle-aged women (*n* = 578; 49.4%), followed by young adults (*n* = 368; 31.4%) and higher-aged women (*n* = 211; 18.0%). Elderly women (*n* = 10; 0.9%) and children and adolescents (*n* = 4; 0.3%) contributed minimally. Temporal distribution showed two major peaks: 2017–2018 and 2022–2023, with invasive carcinoma sharply rising in young and middle-aged women during these periods. A transient decline was observed in 2019–2020 across most age groups. DCIS and papillary carcinoma occurred predominantly among young and middle-aged women, whereas metastatic carcinoma was distributed mainly between young and middle-aged groups, with no cases in the elderly.

Chi-square test of independence revealed a significant association between age group and malignant lesion type (χ², df = 16, *p* < 0.001), confirming that the distribution of malignancies was dependent of age. Logistic regression modeling further demonstrated that both age group and year of diagnosis were significant predictors of invasive carcinoma (*p* < 0.01). Compared with young adults, middle-aged women had significantly higher odds of presenting with invasive carcinoma (OR = 1.8, 95% CI: 1.3–2.6), while higher-aged women also showed increased risk (OR = 1.2, 95% CI: 1.0–1.6). In contrast, children and adolescents and elderly women had markedly reduced odds (OR < 0.2, 95% CI: 0.05–0.3). A temporal effect was observed, with the odds of invasive carcinoma increasing significantly after 2016, peaking in 2017–2018 and again in 2022–2023 (*p* < 0.01).

Descriptive analysis revealed that invasive ductal carcinoma (IDC) was the significantly predominant invasive carcinoma subtype, constituting the majority of cases across all study years (Fig. 6; Supplementary Table 6). IDC incidence rose sharply after 2017, peaking at 301 cases in 2023, with the highest frequencies consistently observed among young adult and middle-aged women (Supplementary Table). Higher-aged patients contributed a modest but increasing proportion from 2018 onward, whereas children and adolescents and elderly women accounted for very few cases overall. Invasive lobular carcinoma (ILC), medullary carcinoma, and mucinous carcinoma were rare, first appearing in 2017 and persisting at low frequencies through 2023, with a distribution concentrated in young adult and middle-aged groups.

Inferential analysis demonstrated a significant association between invasive carcinoma subtype and age group (χ², *p* < 0.001). IDC was disproportionately frequent among young adults and middle-aged women, whereas the rarer histology showed no consistent age-specific predilection. A chi-square test for trend confirmed a significant temporal rise in IDC across the nine-year period (*p* < 0.001), while no consistent increase was observed for lobular, medullary, or mucinous carcinoma. Logistic regression further indicated that young adults and middle-aged patients had significantly higher odds of IDC compared with elderly patients (OR > 6.0, 95% CI 2.5–14.0, *p* < 0.001), independent of year and hospital source.

### Distribution of breast cancer subtypes across age-groups

**Fig. 7:**
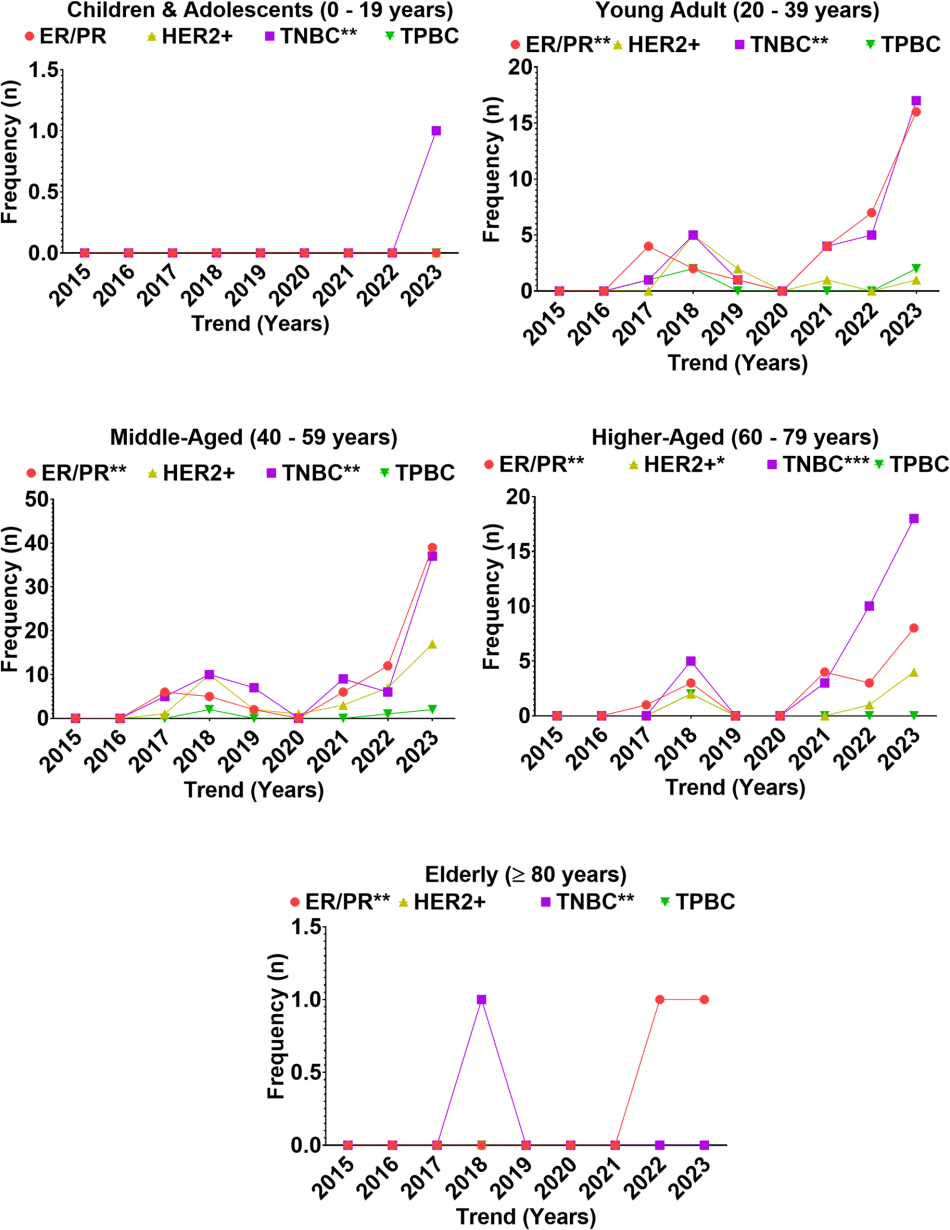
Trends in the occurrence of breast cancer subtypes among age groups of Nigerian women from 2015 to 2023.

The temporal distribution of breast cancer molecular subtypes from 2015 to 2023 revealed marked age-specific variation among Nigerian women (Fig. 7). Among the 1,411 histologically confirmed malignant breast cancers, only 337 (23.88%) underwent immunohistochemical (IHC) profiling for molecular classification (Supplementary Table 7). Across all cases, triple-negative breast cancer (TNBC) was the most prevalent subtype (42.8%), followed by ER/PR-positive tumors (36.9%), HER2-positive disease (16.8%), and TPBC (3.5%) (Supplementary Table 8). Subtype frequencies varied substantially by age. Children and adolescents (≤ 19 years) contributed minimally, with only one TNBC case identified in 2023 and no ER/PR, HER2+, or TPBC tumors, underscoring the negligible burden in this group. By contrast, young adults (20–39 years) exhibited an emerging profile of molecular heterogeneity after 2017, rising steadily through 2023 with 16 ER/PR-positive cases, 17 TNBC, one HER2+, and two TPBC. In this group, TNBC and ER/PR-positive tumors predominated, reflecting an early onset of both aggressive and hormone-sensitive phenotypes.

The middle-aged group (40–59 years) carried the heaviest burden, accounting for the majority of TNBC, ER/PR, and HER2 + tumors. By 2023, ER/PR-positive (*n* = 39) and TNBC (*n* = 37) reached their peaks, alongside 17 HER2 + and 2 TPBC cases, highlighting substantial molecular heterogeneity. Higher-aged women (60–74 years) showed a moderate but rising incidence, peaking in 2023 with 8 ER/PR-positive, 18 TNBC, and 4 HER2 + cases, again with TNBC as the dominant subtype. Elderly women (≥ 75 years) contributed minimally, with only two ER/PR-positive and one TNBC case across the nine-year period. Age-stratified trend analysis further confirmed a sharp increase in TNBC and ER/PR-positive tumors after 2017, with the steepest rise observed in 2023 across all groups except children and adolescents and the elderly.

Inferential statistics supported these descriptive trends. A chi-square test demonstrated a significant association between age group and subtype distribution (χ², *p* < 0.001), with young adults and middle-aged women driving most of the variation. Logistic regression showed that middle-aged women had significantly higher odds of TNBC compared with young adults (OR ≈ 2.0; 95% CI: 1.5–2.7) and were also most likely to present with ER/PR-positive tumors (OR ≈ 2.3; 95% CI: 1.6–3.2). Higher-aged women exhibited moderately elevated odds of TNBC (OR ≈ 1.4; 95% CI: 1.0–2.0), while HER2-positive disease clustered disproportionately in the middle-aged group (OR ≈ 2.5; 95% CI: 1.3–4.7). Children and adolescents and the elderly had negligible odds across all subtypes, with ORs approaching zero. Collectively, these findings identify middle-aged women as the epicenter of molecular heterogeneity, with TNBC and ER/PR-positive cancers dominating, while young adults emerge as a high-risk group for early TNBC onset. Temporal patterns suggest that shifts in environmental exposures, lifestyle changes, or diagnostic practices may underlie the sharp increases observed after 2017.

## Discussion

This research offers important findings on the pathological and clinical features of breast lesions across age groups of Nigerian women, identifying key factors linked to cancer development. Analysis of 3,263 cases showed nearly equal proportions of malignant and benign tumors. The study reveals that tumor location (laterality), number of children (parity), menopause status, tumor grade, and biopsy method are significant predictors of breast cancer risk in this population.

Analysis of geographical distribution indicated no statistically significant difference in malignancy risk across regions. Although patients from the east and west had slightly higher frequencies of malignancy, these associations were not statistically significant. This suggests that breast cancer is a nationwide burden in Nigeria, transcending regional boundaries^7,28^. The breast lesion collection method depicted that excision biopsy was the most common method of breast lesion collection, with 64.94% of the cases performed in young adults and 27.37% in middle-aged individuals. Isikhuemen et al.^29^, reported that high excision biopsy rates are caused by high incidence of fibroadenomas, which is highly significant among the young adults. Likewise, this high occurrence among the young adults could be as a result of early detection in younger populations due to their increased awareness and healthcare access. This finding correlates with the reports of Effiong et al.^11^ and Effiong et al.^7^ which indicated a higher breast cancer knowledge among young adults compared to middle-aged individuals. Findings by Alfadul et al.^30^, Anakwenze et al.^31^, Petrova et al.^32^, Wassie et al.^33^, Alfadul et al.^30^, Agodirin et al.^27,34^ reported lower awareness and late presentation among higher-aged and elderly women, this could account for lower excision biopsy rates observed among this age group. Mastectomy involves the partial or complete removal of the breast with the aim of treating breast cancer. The data showed that the frequency of mastectomy in the middle-aged individuals was higher compared to other age groups. This results are similar to the reports by Bert et al.^35^, Lu et al.^36^, Xie et al.^37^; Boniface et al.^38^; Dayaratna et al.^39^.

With respect to menopausal status, malignancy was significantly more prevalent among menopausal and postmenopausal women. Postmenopausal women had almost five times the odds of having malignant lesions compared to premenopausal women (OR: 4.82; 95% CI: 3.33–6.97; *p* < 0.001), and similar associations were seen with menopausal women (OR: 2.73; 95% CI: 2.21–3.38; *p* < 0.001). This is consistent with the hormonal theory of breast carcinogenesis, where estrogen exposure across a lifetime plays a crucial role^40^. A study conducted in Lagos State, Nigeria, also reported a higher prevalence of breast cancer among postmenopausal women, underscoring the need for targeted screening strategies in older age groups^41^. Similarly, parity showed a strong correlation with malignancy. The data revealed that women with higher parity (≥ 3 children) were more likely to develop breast cancer. However, this finding diverges from reports in Western populations where high parity is typically protective. This results also negates the findings by Adebamowo et al.^42^ a case-control study in Nigeria found that high parity and long duration of breastfeeding are associated with reduced risk of breast cancer, for every 12 months of breastfeeding, the risk of breast cancer dropped by 7%.

Tumor grade distribution varied with age, with Grade 2 predominating across groups and middle-aged patients carrying the highest burden. Higher-aged individuals showed more Grade 3 tumors, suggesting greater aggressiveness with age, while Grade 1 tumors were rare overall. Similar findings in Nigeria by Ekanem and Aligbe^43^ and Ethiopia by Hadgu et al.^44^ suggest high-grade, aggressive breast cancers are common in African women, likely due to late diagnosis and limited screening. Early detection strategies are urgently needed in sub-Saharan Africa to address this burden.

Family history is a major risk factor of breast cancer which aids to inform treatment and prevention of the disease^25^. The family history data recorded was of a low proportion, which is very concerning due to the vital role family history plays in breast cancer. This further reveals a gap in record keeping and patient recording, also presented a limitation of this study. The lump duration was also taken into account. Most patients had breast lumps for less than a year before presentation, similar to reports by Nimbalkar et al.^45^ however, a small percentage of patients had lumps persisting for over 5 years, spanning to 30 years which highlights the need for stronger public health enlightenment and screening programs for early diagnosis.

Benign lesions are lesions that are not cancerous. There were numerous benign breast lesions identified based on their histopathological features. There was a higher proportion of benign cases (56.76%) compared to the malignant cases (43.24%). Benign cases were highest among the young-adults, and least among the middle-aged, indicating the vital role age plays as a risk factor of breast cancer. These benign conditions among the younger aged tend to resolve without invasive treatments. Likewise, the decrease in the occurrence of benign cases with age highlights the need for high vigilances as benign breast lumps could potentially mask or co-exist with malignant lumps. Fibroadenoma was found to be the most common benign lesion that accounted for 43.52% of all benign cases, with the intracanalicular pattern being the most common subtype (43.60%). This result is consistent with global trends reported by Gupta et al.^46^; Pandit et al.^47^; Salati et al.^48^. The incidence of fibroadenoma decreased with age which could be attributed to hormonal changes during puberty and early adulthood, and the regression in hormonal levels after menopause. Fibrocystic change, was the second most common benign condition. It was observed to have followed a similar trend of decreasing with increasing age, as reported by Dev et al.^49^. This trend was also similar in the occurrence of adenosis and phylloides lesion. However, the occurrence of chronic inflammatory benign lumps were very prominent in the middle-aged and higher-aged. This possibly reflects underlying chronic inflammatory processes among these age groups^50^. The disparity in the distribution of benign breast lesions across age groups highlights the importance of age-specific diagnosis and treatment approaches in managing benign breast diseases in Nigeria.

Malignant lesions are cancerous in nature. There was no significant difference in the occurrence of malignant cases compared to benign cases among the patients, although, the malignant cases were lower than the benign cases. The occurrence of malignant cases increased with age, from children and adolescents to middle-aged, after which a sharp decline was observed between the higher-aged and the elderly. The decline in malignant case incidence among the older women, after increasing through middle age, may be due to age-associated loss of cellular regenerative capacity, which diminishes the likelihood of uncontrolled cancerous growth in elderly tissues^6^. Likewise, the high prevalence of malignancies among the middle-aged implies the need for early detection, improved awareness and the prioritization of regular diagnostic and screening services for advanced women^51^. Invasive carcinoma was the most common type of malignant lesion with a percentage of 84.62%, similar to findings by Bosompem et al.^52^, with a percentage of 97.10%. The most common subtype is invasive carcinoma of no special kind (ductal), accounting for 87.86% of cases. This high prevalence is attributed to factors such as delayed diagnosis and limited access to early screening programs, which contribute to the advanced stage of breast cancer. Other factors include genetic predisposition, environmental exposures, and reproductive history. Ductal carcinoma in situ (DCIS), which makes up 2.34% of all malignant lesions, affects only the ducts. Early detection with mammographic screening can improve prognosis and delay progression to invasive disease. However, in developing countries like Nigeria, screening programs are not widely available, leading to a lower diagnosis rate for non-invasive cancers like DCIS. Middle-aged women have the highest incidence of bilateral breast cancer and invasive carcinoma, followed by young adults, the elderly, and children and adolescents. These age-specific tendencies is crucial for creating successful screening and prevention programs.

Breast cancer is a heterogeneous disease with distinct molecular subtypes, including triple-negative breast cancer (TNBC), estrogen receptor-positive/progesterone receptor-positive (ER+/PR+), human epidermal growth factor receptor 2-positive (HER2+), and triple-positive breast cancer (TPBC)^53^. The present study revealed that TNBC was the most prevalent subtype, accounting for 42.77% of all immunohistochemically classified cases. This high occurrence is of clinical concern due to TNBC’s aggressive behavior, limited treatment options, and higher risk of recurrence. Notably, TNBC was the only subtype observed among children and adolescents, highlighting the alarming potential for early-onset aggressive breast cancer. These findings are consistent with previous reports from Nigeria and other Sub-Saharan African countries^52^, as well as broader epidemiological studies showing higher TNBC prevalence among women of African descent. ER+/PR + breast cancer was the second most common subtype, constituting 36.87% of cases. This subtype is known to respond well to hormonal therapies such as tamoxifen and aromatase inhibitors, improving prognosis and reducing recurrence risk^54^. The predominance of ER+/PR + among young and middle-aged adults observed in this study supports the notion that endogenous hormonal factors around reproductive age may contribute to tumor development. These findings align with global patterns and corroborate prior studies^55^. HER2 + and TPBC subtypes were less common, accounting for 16.81% and 3.54% respectively. Although HER2 + breast cancer is known for its aggressive nature, the availability of HER2-targeted therapies such as trastuzumab (Herceptin) has significantly improved outcomes^56^. The lower prevalence of HER2 + in this cohort mirrors earlier findings from Nigerian populations^57^. In terms of age distribution, middle-aged women (40–59 years) bore the greatest burden of all subtypes, particularly TNBC and ER+/PR+. A significant increase in these subtypes in 2023 suggests either improved diagnostic capacity or a genuine rise in disease incidence. Meanwhile, young adults (20–39 years) showed a steady increase in ER+/PR + and TNBC cases, emphasizing the need for early detection and tailored intervention strategies. Breast cancer was rare in women aged ≥ 80 years, but occasional TNBC and HER2 + cases still occurred, indicating continued albeit lower risk in this demographic. Overall, the data underscores the importance of subtype-specific breast cancer surveillance and the need for expanded access to molecular diagnostics and targeted therapies to address the aggressive disease patterns in Nigerian women.

## Conclusion

This study offers valuable insights into the age-related patterns of breast cancer in Nigeria, revealing significant variations in breast lesion types and molecular subtypes across different age groups. Middle-aged women (40–59 years) showed the highest prevalence of malignant lesions, particularly invasive carcinoma, while young adults (20–39 years) more frequently presented with benign lesions such as fibroadenoma and bilateral benign tumors. These findings underscore the importance of targeted public health interventions tailored to the needs of specific age groups, particularly middle-aged and young adult Nigerian women. Moreover, the predominance of triple-negative breast cancer among middle-aged women highlights the urgency of developing age- and subtype-specific preventive and therapeutic strategies.

However, this study has important limitations. A substantial proportion of records lacked key clinical information, 69.41% had no lump duration and molecular classification was available for only 24.03%, which restricts the reliability and generalizability of the conclusions. The very low reported prevalence of family history (1.23%) also reflect underreporting rather than true incidence. Furthermore, the observed decline in diagnoses between 2019 and 2021, followed by a sharp rise in 2023, likely reflects healthcare disruptions during the COVID-19 pandemic and subsequent delayed presentations, rather than a true epidemiological shift. Finally, findings for the elderly group (≥ 80 years) should be regarded as exploratory due to the very small sample size (*n* = 11), limiting the reliability of conclusions in this subgroup. Recognizing these limitations is essential for interpreting the findings and for guiding future research that addresses data completeness, subgroup representation, and pandemic-related effects on cancer detection and care.

## Supplementary Information

Below is the link to the electronic supplementary material.

Supplementary Material 1

## Data Availability

The data and materials used in this study are available as supplementary files, further request can be made to the corresponding author.
