# Supplementary material for: Age-specific patterns of breast cancer in Nigerian women unraveled through histological analysis
Source: Sci Rep. 2025 Nov 29;16:128. doi: 10.1038/s41598-025-28685-0 (PMC12764456; doi:10.1038/s41598-025-28685-0)
Supplement: Supplementary file 1 — Supplementary Material 1 [file 41598_2025_28685_MOESM1_ESM.docx]

Supplementary Table 1: Distribution of Malignant and Benign Lesions Across Age Groups

| **Age group** | **Malignant n (%)** | **Benign n (%)** | **Total n** |
| --- | --- | --- | --- |
| Children and adolescents | 7 (1.7) | 395 (98.3) | 402 |
| Young Adults | 438 (29.2) | 1062 (70.8) | 1500 |
| Middle-aged | 752 (68.1) | 352 (31.9) | 1104 |
| Higher-aged | 203 (82.5) | 43 (17.5) | 246 |
| Elderly | 11 (100.0) | 0 (0.0) | 11 |
| **Total** | **1411 (45.6)** | **1852 (54.4)** | **3263** |

Supplementary Table 2: Yearly Distribution of Malignant and Benign Lesions Across Age Groups

| **Children and adolescents** | |  |  |
| --- | --- | --- | --- |
| Year | Malignant n (%) | Benign n (%) | Total n |
| 2015 | 0 (0.0) | 19 (100.0) | 19 |
| 2016 | 0 (0.0) | 64 (100.0) | 64 |
| 2017 | 0 (0.0) | 62 (100.0) | 62 |
| 2018 | 0 (0.0) | 58 (100.0) | 58 |
| 2019 | 0 (0.0) | 38 (100.0) | 38 |
| 2020 | 0 (0.0) | 22 (100.0) | 22 |
| 2021 | 0 (0.0) | 30 (100.0) | 30 |
| 2022 | 2 (3.7) | 52 (96.3) | 54 |
| 2023 | 5 (9.1) | 50 (90.9) | 55 |
| Total | 7 (1.7) | 395 (98.3) | 402 |
|  |  |  |  |
| Young Adults | |  |  |
| Year | Malignant n (%) | Benign n (%) | Total n |
| 2015 | 10 (21.3) | 37 (78.7) | 47 |
| 2016 | 14 (8.8) | 146 (91.2) | 160 |
| 2017 | 68 (29.1) | 166 (70.9) | 234 |
| 2018 | 96 (32.8) | 197 (67.2) | 293 |
| 2019 | 31 (23.3) | 102 (76.7) | 133 |
| 2020 | 17 (26.2) | 48 (73.8) | 65 |
| 2021 | 45 (30.8) | 101 (69.2) | 146 |
| 2022 | 71 (33.5) | 141 (66.5) | 212 |
| 2023 | 86 (41.0) | 124 (59.0) | 210 |
| Total | 438 (29.2) | 1062 (70.8) | 1500 |
|  |  |  |  |
| Middle-aged | |  |  |
| Year | Malignant n (%) | Benign n (%) | Total n |
| 2015 | 13 (50.0) | 13 (50.0) | 26 |
| 2016 | 22 (33.8) | 43 (66.2) | 65 |
| 2017 | 103 (65.2) | 55 (34.8) | 158 |
| 2018 | 123 (68.7) | 56 (31.3) | 179 |
| 2019 | 57 (68.7) | 26 (31.3) | 83 |
| 2020 | 19 (52.8) | 17 (47.2) | 36 |
| 2021 | 89 (77.4) | 26 (22.6) | 115 |
| 2022 | 125 (69.8) | 54 (30.2) | 179 |
| 2023 | 201 (76.4) | 62 (23.6) | 263 |
| Total | 752 (68.1) | 352 (31.9) | 1104 |
|  |  |  |  |
| Higher-aged | |  |  |
| Year | Malignant n (%) | Benign n (%) | Total n |
| 2015 | 3 (60.0) | 2 (40.0) | 5 |
| 2016 | 6 (100.0) | 0 (0.0) | 6 |
| 2017 | 20 (87.0) | 3 (13.0) | 23 |
| 2018 | 36 (83.7) | 7 (16.3) | 43 |
| 2019 | 14 (73.7) | 5 (26.3) | 19 |
| 2020 | 4 (80.0) | 1 (20.0) | 5 |
| 2021 | 24 (85.7) | 4 (14.3) | 28 |
| 2022 | 35 (70.0) | 15 (30.0) | 50 |
| 2023 | 61 (91.0) | 6 (9.0) | 67 |
| Total | 203 (82.5) | 43 (17.5) | 246 |
|  |  |  |  |
| Elderly |  |  |  |
| Year | Malignant n (%) | Benign n (%) | Total n |
| 2015 | 1 (100.0) | 0 (0.0) | 1 |
| 2016 | 1 (100.0) | 0 (0.0) | 1 |
| 2017 | 0 (0.0) | 0 (0.0) | 0 |
| 2018 | 3 (100.0) | 0 (0.0) | 3 |
| 2019 | 0 (0.0) | 0 (0.0) | 0 |
| 2020 | 1 (100.0) | 0 (0.0) | 1 |
| 2021 | 1 (100.0) | 0 (0.0) | 1 |
| 2022 | 3 (100.0) | 0 (0.0) | 3 |
| 2023 | 1 (100.0) | 0 (0.0) | 1 |
| Total | 11 (100.0) | 0 (0.0) | 11 |

Supplementary Table 3: Distribution of malignant lesion nuclear grades across age groups with odds ratios (OR) and 95% confidence intervals (CI)

| **Age Group** | **Grade 1 n (%)** | **Grade 2 n (%)** | **Grade 3 n (%)** | **Total n (%)** | **Odds Ratio (OR)** | **95% CI (Lower–Upper)** |
| --- | --- | --- | --- | --- | --- | --- |
| Children & Adolescents | 0 (0.0) | 3 (75.0) | 1 (25.0) | 4 (0.6) | 3.00 | 0.31 – 28.84 |
| Young Adults | 13 (6.1) | 134 (63.2) | 65 (30.7) | 212 (29.9) | 0.57 | 0.06 – 5.60 |
| Middle-aged | 7 (1.8) | 308 (79.2) | 74 (19.0) | 389 (54.9) | 1.27 | 0.13 – 12.35 |
| Higher-aged | 10 (10.0) | 50 (50.0) | 40 (40.0) | 100 (14.1) | 0.33 | 0.03 – 3.31 |
| Elderly | 0 (0.0) | 3 (75.0) | 1 (25.0) | 4 (0.6) | 1.00 | 0.04 – 24.55 |
| **Total** | 30 (4.2) | 498 (70.2) | 181 (25.5) | 709 (100) | — | — |

Supplementary Table 4: Distribution of benign breast lesions across age groups

| **Lesion type** | **Children & Adolescents**  **n (%)** | **Young Adults n (%)** | **Middle-aged n (%)** | **Higher-aged n (%)** | **Elderly**  **n (%)** | **Total**  **n (%)** |
| --- | --- | --- | --- | --- | --- | --- |
| **Adenosis** | 17 (16.7) | 54 (52.9) | 22 (21.6) | 0 (0.0) | 0 (0.0) | 93 (100) |
| **Fibrosis** | 35 (8.1) | 275 (63.5) | 124 (28.6) | 16 (3.7) | 0 (0.0) | 433 (100) |
| **Fibroadenoma** | 270 (31.5) | 446 (52.1) | 78 (9.1) | 12 (1.4) | 0 (0.0) | 856 (100) |
| **Chronic inflammation** | 1 (2.0) | 26 (52.0) | 14 (28.0) | 6 (12.0) | 0 (0.0) | 47 (100) |
| **Phyllodes tumor** | 10 (15.6) | 34 (53.1) | 15 (23.4) | 0 (0.0) | 0 (0.0) | 64 (100) |

Supplementary Table 5: Distribution of malignant breast lesions across age groups

| **Carcinoma subtype** | **Children and adolescents n (%)** | **Young Adults**  **n (%)** | **Middle-aged**  **n (%)** | **Higher-aged**  **n (%)** | **Elderly**  **n (%)** | **Total**  **n (%)** |
| --- | --- | --- | --- | --- | --- | --- |
| **Invasive carcinoma** | 4 (0.4) | 368 (35.3) | 578 (55.5) | 211 (20.3) | 10 (1.0) | 1,171 (100) |
| **Ductal carcinoma in situ (DCIS)** | 0 (0.0) | 19 (46.3) | 38 (46.3) | 3 (7.3) | 1 (2.4) | 41 (100) |
| **Malignant phyllodes** | 0 (0.0) | 4 (50.0) | 2 (25.0) | 2 (25.0) | 0 (0.0) | 8 (100) |
| **Metastatic carcinoma** | 0 (0.0) | 11 (45.8) | 14 (58.3) | 0 (0.0) | 0 (0.0) | 24 (100) |
| **Papillary carcinoma** | 0 (0.0) | 7 (58.3) | 6 (50.0) | 2 (16.7) | 0 (0.0) | 12 (100) |

Supplementary Table 6: Distribution of invasive carcinoma subtypes across age groups

| **Histologic subtype** | **Children and adolescents**  **n (%)** | **Young Adults n (%)** | **Middle-aged n (%)** | **Higher-aged n (%)** | **Elderly**  **n (%)** | **Total**  **n (%)** |
| --- | --- | --- | --- | --- | --- | --- |
| Invasive ductal carcinoma | 4 (0.5) | 314 (34.6) | 557 (61.4) | 166 (18.3) | 8 (0.9) | 1,049 (100) |
| Invasive lobular carcinoma | 0 (0.0) | 6 (23.1) | 13 (50.0) | 6 (23.1) | 0 (0.0) | 25 (100) |
| Medullary carcinoma | 0 (0.0) | 9 (37.5) | 14 (58.3) | 2 (8.3) | 0 (0.0) | 24 (100) |
| Mucinous carcinoma | 0 (0.0) | 10 (47.6) | 8 (38.1) | 3 (14.3) | 0 (0.0) | 21 (100) |

Supplementary Table 7: Yearly distribution of breast cancer molecular subtypes across age groups (2015–2023)

| **Year** | **Children & Adolescents**  **n (%)** | **Young Adults**  **n (%)** | **Middle-aged n (%)** | **Higher-aged**  **n (%)** | **Elderly**  **n (%)** | **Total**  **n (%)** |
| --- | --- | --- | --- | --- | --- | --- |
| 2015 | 0 (0.0) | 6 (28.6) | 7 (33.3) | 3 (14.3) | 1 (4.8) | 17 (100) |
| 2016 | 0 (0.0) | 5 (21.7) | 12 (52.2) | 5 (21.7) | 1 (4.3) | 23 (100) |
| 2017 | 0 (0.0) | 7 (17.5) | 20 (50.0) | 10 (25.0) | 3 (7.5) | 40 (100) |
| 2018 | 0 (0.0) | 6 (13.6) | 28 (63.6) | 9 (20.5) | 1 (2.3) | 44 (100) |
| 2019 | 0 (0.0) | 12 (23.1) | 23 (44.2) | 15 (28.8) | 2 (3.8) | 52 (100) |
| 2020 | 0 (0.0) | 8 (22.9) | 19 (54.3) | 7 (20.0) | 1 (2.9) | 35 (100) |
| 2021 | 0 (0.0) | 9 (22.5) | 19 (47.5) | 11 (27.5) | 1 (2.5) | 40 (100) |
| 2022 | 0 (0.0) | 10 (22.7) | 22 (50.0) | 11 (25.0) | 1 (2.3) | 44 (100) |
| 2023 | 1 (3.0) | 16 (48.5) | 21 (63.6) | 7 (21.2) | 2 (6.1) | 47 (100) |
| **Total** | 1 (0.3) | 79 (23.4) | 171 (50.7) | 78 (23.1) | 13 (3.9) | 337 (100) |

Supplementary Table 8: Distribution of breast cancer molecular subtypes across age groups (2015–2023)

| **Age Group** | **ER/PR n (%)** | **TNBC n (%)** | **HER2+ n (%)** | **TPBC n (%)** | **Total n (%)** |
| --- | --- | --- | --- | --- | --- |
| Children & Adolescents | 0 (0.0) | 1 (100.0) | 0 (0.0) | 0 (0.0) | 1 (0.2) |
| Young Adults | 34 (43.0) | 33 (41.8) | 9 (11.4) | 3 (3.8) | 79 (14.2) |
| Middle-aged | 70 (40.9) | 74 (43.3) | 41 (24.0) | 5 (2.9) | 171 (30.8) |
| Higher-aged | 19 (34.6) | 36 (65.5) | 7 (12.7) | 2 (3.6) | 55 (9.9) |
| Elderly | 2 (66.7) | 1 (33.3) | 0 (0.0) | 0 (0.0) | 3 (0.5) |
| **Total** | 125 (41.3) | 145 (47.9) | 57 (18.8) | 10 (3.3) | 337 (100) |
